# A Census of Human Methionine-Rich Prion-like Domain-Containing Proteins

**DOI:** 10.3390/antiox11071289

**Published:** 2022-06-29

**Authors:** Juan Carlos Aledo

**Affiliations:** Department of Molecular Biology and Biochemistry, University of Malaga, 29071 Malaga, Spain; caledo@uma.es

**Keywords:** liquid–liquid phase separation, low complexity region, methionine, prion, protein domain

## Abstract

Methionine-rich prion-like proteins can regulate liquid–liquid phase separation processes in response to stresses. To date, however, very few proteins have been identified as methionine-rich prion-like. Herein, we have performed a computational survey of the human proteome to search for methionine-rich prion-like domains. We present a census of 51 manually curated methionine-rich prion-like proteins. Our results show that these proteins tend to be modular in nature, with molecular sizes significantly greater than those we would expect due to random sampling effects. These proteins also exhibit a remarkably high degree of spatial compaction when compared to average human proteins, even when protein size is accounted for. Computational evidence suggests that such a high degree of compactness might be due to the aggregation of methionine residues, pointing to a potential redox regulation of compactness. Gene ontology and network analyses, performed to shed light on the biological processes in which these proteins might participate, indicate that methionine-rich and non-methionine-rich prion-like proteins share gene ontology terms related to the regulation of transcription and translation but, more interestingly, these analyses also reveal that proteins from the methionine-rich group tend to share more gene ontology terms among them than they do with their non-methionine-rich prion-like counterparts.

## 1. Introduction

Since the completion of the Human Genome Project in 2003 to the present, we have witnessed an impressive development in the understanding of how the many proteins that make up a proteome contribute to the functional organization of living matter [1,2]. However, we remain largely ignorant about the mechanisms by which certain proteins that contain sequences with gibberish-like low-complexity regions (LCRs) may function. These enigmatic LCRs, present in 10–20% of human proteins [3,4] and accounting for around 0.4% of the amino acid sites in eukaryotic proteomes [5], were originally thought of as junk. In any event, many LCRs are genetically unstable because they facilitate replication slippage or cause recombinational inaccuracies [6], which confers to these sequences a high pathological potential. On the one hand, the uncontrolled expansion of short sequence stretches can generate high amyloidogenicity [7], which is known to be behind a number of developmental and neurodegenerative diseases [8]. On the other hand, single point mutations within LCRs can be disease-causing [9,10,11,12,13], probably because the mutation stabilizes the pathogenic aggregates [14,15].

The high abundance in most proteomes of these prion-like domains (PrLDs) with low sequence complexity [3,4] and, in many instances, evolutionary conservation through orthologous proteins [5], are difficult to reconcile in light of sequence stretches with no function, but posing a risk for the cell carrying them. Thus, while it is correct that these LCRs can be targets of pathogenic mutations, it is also true that they must perform essential biological functions. Although most of the LCRs present in the known protein universe remain uncharacterized [16], there is growing evidence pointing to a prominent role for these PrLDs in the assembly/disassembly of cellular condensates in response to specific stimuli [3,17]. In this way, the unanticipated finding that recombinant purified LCRs, separately from the rest of the protein, can undergo phase transition from an aqueous solution to form either liquid-like droplets or hydrogels [18,19,20], has placed these low sequence complexity domains in the focus of interest. Thus, overcoming the original reluctance to consider these sequences to be of biological relevance, prion-like low-complexity sequences are currently thought to be key regulators of protein solubility and phase behavior [21].

The ability of certain proteins, known as scaffolds, to form transient productive intra- and intermolecular interactions is behind the formation of specific but non-stoichiometric supramolecular structures (quinary structures) that give rise to biomolecular condensates, often called membraneless organelles (MLOs). These MLOs are dynamic structures quickly assembled and disassembled in response to changing conditions, through a reversible process involving demixing into two distinct liquid phases, referred to as liquid–liquid phase separation (LLPS) [22]. Prion-like low complexity sequences may play a relevant role in LLPS, providing a combination of organizational specificity and dynamic flexibility, necessary for an adequate response to stimuli. In this respect, post-translational modifications of the residues present in these LCRs offer a convenient strategy to reshape the residue–residue interaction networks that determine the dynamics of LLPS [17].

In this context, recent works have identified reversible methionine oxidation as a redox sensor involved in the dynamic assembly/disassembly of biomolecular condensates [20,23,24]. Thus, yeast ataxin-2 has been reported to form intracellular condensates required for proper cellular signaling in response to nutrient availability [20]. Inspection of the yeast ataxin-2 LCR involved into self-association and phase separation revealed a striking enrichment in methionine residues. Furthermore, these authors showed that ataxin-2 liquid-like droplets exposed to hydrogen peroxide melted in a fully reversible manner. Thus, the addition of methionine sulfoxide reductase enzymes, under conditions favorable to reduce oxidized methionine residues back to methionine, fully restore the ability of ataxin-2 to condensate and form droplets [23]. Interestingly, liquid-like droplets made from mutated variants of yeast ataxin-2 bearing methionine-to-tyrosine (or phenylalanine) substitutions were resistant to H_2_O_2_-mediated melting, pointing to reversible methionine oxidation as an additional means of regulating PrLD-LCRs [23]. Shortly after this finding, Lin et al. reported that a methionine-rich (MR) low-complexity domain, located within the C-terminal region of the Tar DNA-binding protein 43 (TDP-43), formed a redox switch able to regulate the self-association of the protein to form labile cross-β polymers and liquid-like droplets. Thus, as in the case of the yeast ataxin-2, experimental evidence suggests that the MR-PrLD from the human TDP-43 protein functions as a redox sensor [24].

Despite the suitability of the methionine side chain for protein-protein interactions, its biological potential has been largely overlooked [25]. In this work, we have computationally explored the human proteome in search of proteins containing MR-PrLDs that may fulfil a redox sensor function.

## 2. Materials and Methods

### 2.1. Dataset

The human reference proteome was obtained from UniProt [26]. Subsequently, we filtered out those peptides with less than 100 residues, a process that yielded a collection of 19,636 different human proteins.

### 2.2. Gene Ontology Enrichment Analyses

The group of human proteins without methionine (other than the initiation methionine) in their sequences were subjected to GO term enrichment analyses using the Gene Ontology Consortium website [27] and the analysis tool from the PANTHER Classification System [28]. The analyses were performed using a reference list formed by all Homo sapiens genes in the database (PANTHER 16.0). A hypergeometric test with Bonferroni correction for multiple comparisons was used to select significantly (*p*-valued < 10^−6^) enriched terms. Fold-enrichment was computed as the ratio of the number of proteins annotated with the GO term in the test set to the number of proteins annotated with said term in the reference (whole human proteome) set.

### 2.3. Spatial Methionine Pattern Analysis

Spatial point pattern analysis is an approach commonly used in ecology to examine the spatial distribution of clustered, dispersed, or randomly occurring individual organisms within a given region [29]. Herein, we have adopted such an approach to study the distribution of methionine residues along the primary structure of proteins. Regardless of the methionine abundance for a given protein, the many or few methionine residues present in that protein can be randomly distributed throughout the primary structure or, alternatively, they may exhibit some degree of tendency either to cluster together or to disperse, in response to structural/functional requirements. To determine when the methionine residues from a given protein were distributed unevenly across its primary structure, we adopted a transect-based method where the protein length was divided into segments of 50 residues and in each of these segments, the number of methionine residues present was computed. In this way, for each protein, we computed the coefficient of variation, given by the quotient (*q*) of the standard deviation between the mean:

(1) $$q=\frac{\sqrt{N\;\sum_{i=1}^{N}{\left(x_{i}-\frac{1}{N}\sum_{i=1}^{N}x_{i}\right)}^{2}}}{\sum_{i=1}^{N}x_{i}}$$

where, *N* is the number of segments and $x_{i}$ is the number of methionine residues in the *i*-th segment. A segment length of 50 residues was chosen because the average methionine abundance is around 2%, which means that the expected number of methionine residues in 50-residue-long segments, when they are randomly distributed according to a Poisson distribution, is 1. For a Poisson (random) distribution where the expectation and variance are equal, we expect a coefficient of variation value close to 1. For this reason, the coefficient of variation has often been used as an aggregation index [30].

### 2.4. Empirical Null Distributions

For proteins in which their methionine residues are randomly distributed according to a Poisson distribution, values of the coefficient of variation are expected to be around 1. Thus, values much greater than 1 (aggregation) or much less than 1 (dispersion) may be attributed to structural/functional causes behind the departure from the random distribution of methionine residues. However, some degree of deviation from the value of 1 could just be due to chance. Furthermore, the protein size and methionine abundance, which vary from protein to protein, can influence the random distribution (Figures S1 and S2, respectively). That is, they can lead to non-Poisson random distributions. Therefore, to determine the *q* threshold levels that discriminate between clustered, dispersed, and random distributions, we resorted to empirical null distribution to assess the degree of clustering/dispersion and its statistical significance. 

**Figure 1:**
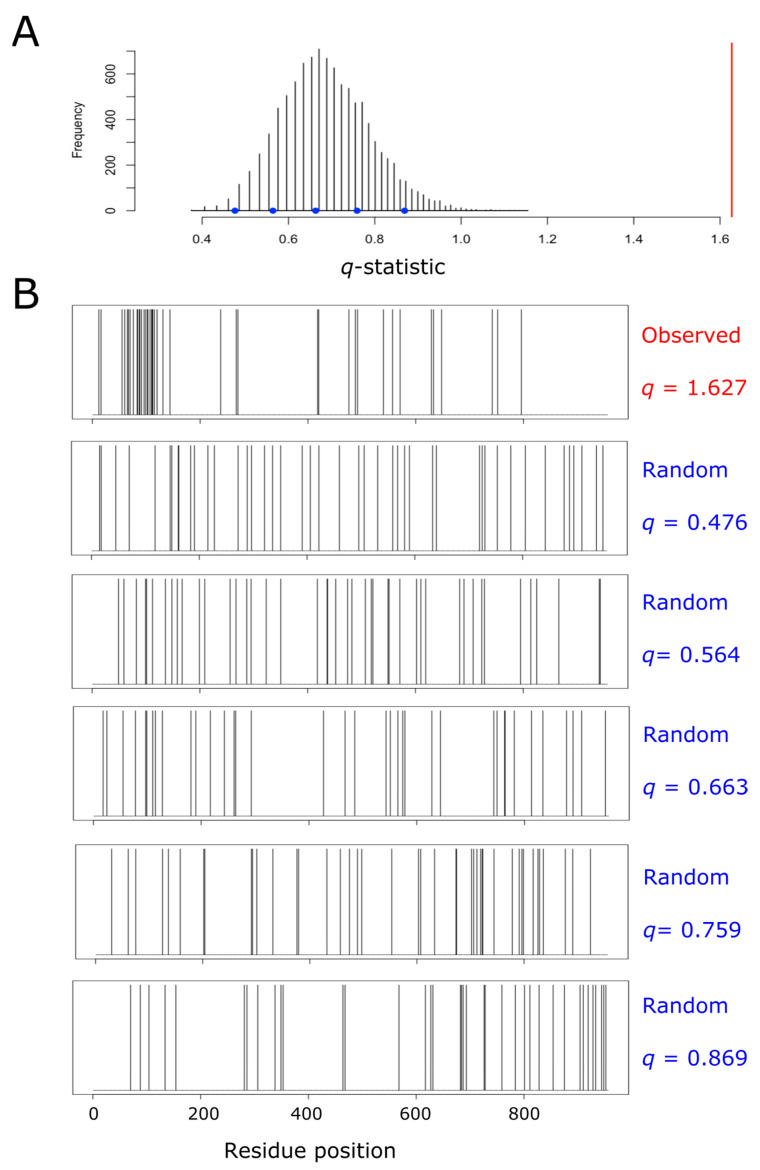
Empirical null distribution of the *q*-statistic for the protein pre-mRNA-processing factor 40 homolog A. This protein (O75400, UniProt ID) has been taken as an example to illustrate the process leading to the detection of methionine residue clustering, and the subsequent labeling of the protein as a member of the ‘aggregation’ category. The protein, which is 956 amino acids long, contains 42 methionyl residues (once the initiation methionine has been removed). Thus, we randomly distributed 42 methionine residues across a sequence of 956 amino acids, and computed the resulting value of the coefficient of variation (*q*). This process was repeated 10,000 times, and the resulting distribution of the statistic *q* was plotted (**A**). The spatial distribution of methionine residues across the protein sequence is shown in (**B**). The top plot from (**B**) correspond to the real observed distribution for the protein being analyzed. The value of *q* computed for this protein was 1.627, and it is indicated by the red vertical line in (**A**). The remaining five plots displayed in (**B**) show the dispositions of methionine across the lineal sequence when randomly distributed at different values of *q*, as indicated by the blue circles in (**A**).

For each single protein, we built its own null distribution, as subsequently described. We started by counting the number of methionine residues (m) and the total length of the protein (N). After that, we created a binary vector containing N–m zeros and m ones at randomly chosen positions. For this vector, the coefficient of variation was computed, as explained above. The process of randomly forming a binary vector and computing its associated *q*-statistic was repeated 10,000 times for each protein. Thus, for each single protein, we were able to plot its own null distribution, thus taking into account the potential effect of protein size and methionine abundance on the distribution of *q*. In this way, all the proteins from the human proteome were sorted into three categories: aggregation, dispersion, and random, using their own empirical null distribution and a significance level of α = 0.0001. As an example, aiming to illustrate the procedure carried out within the entire proteome, Figure 1 shows the null distribution obtained for pre-mRNA-processing factor 40 homolog A (O75400), a protein that fell into the ‘aggregation’ category. 

### 2.5. Computational Definition of MR-PrLD

For the purposes of the current study, we defined an MR-PrLD as any sequence stretch that satisfies the following criteria: (i) the considered sequence must be hosted into a protein whose methionine residues show a clear tendency to cluster together; that is, the stretch must be present in a protein labeled as a member of the ‘aggregation’ group, as defined above. (ii) The sequence stretch must contain an LCR contributed by methionine or methionine and other amino acids. To detect such LCRs, we resorted to fLPS, a program that rapidly annotates single- and multiple-residue biased regions in the sequence being analyzed [31,32]. The annotated sequence returned by this software was subsequently parsed, using an ad hoc R script (*pasefLPS*) to recover only those regions where methionine contributed significantly, *p*-value < 10^−5^, to sequence bias. Furthermore, (iii) this LCR must match with a PrLD, as predicted by a hidden-Markov model algorithm, such as PLAAC [33]. Again, we made use of an R script, *parsePLAAC*, to manage those sequence stretches predicted as PrLD at a threshold probability of 0.9. Finally, (iv) the sequence stretches containing less than 5 methionine residues must be filtered out.

Criteria (ii) and (iii) were simultaneously addressed using the R script *score.mrr*, which calls and manages different scripts (*fLPS, PLAAC, parsefLPS,* and *parsePLAAC*), coordinating the returned outputs and combining them into one single output object, which reveals the positions at which the detected MR-PrLDs start and end, as well as their *p*-values. All the R codes mentioned above can be found at [34].

### 2.6. Compactness Index

For each protein, as an estimate of structural compactness, we determined the interatomic distance between the $C_{\alpha }$ of the N-terminal amino acid and the $C_{\alpha }\;$ of the C-terminal residue. To this end, we used the coordinates obtained from the AlphaFold protein structure database [35,36]. This distance in angstroms (*d*) was subsequently divided by the number of residues (N) of the protein being analyzed, to provide the compactness index, $I_{c}=\frac{d}{N}$. Thus, the lower the *I_c_* value, the more compact the protein. 

It should be pointed out that we failed to obtain from the AlphaFold database a reliable structure for 2 (Q9P2D1 and Q6KC79) of the 51 proteins belonging to the MR-PrLD set. To quantify the significance of the mean *I_c_* computed for the MR-PrLD group, we resorted to constructing empirical distributions. For this purpose, from the whole set of non-membrane proteins, we randomly sampled 49 proteins and computed their mean *I_c_*, repeating the process 10,000 times. The random sampling was performed in three different ways: (i) without any restriction at all, (ii) imposing a matching in protein sizes between the MR-PrLD group and the sampled proteins, and (iii) imposing such a matching, but for the methionine content rather than for protein size. 

### 2.7. Protein Networks Based on GO Terms and Assortativity Analysis

Using data provided by Iglesias and et al. [37] and data generated in the current work, we have established a set of 148 unique human protein-containing PrLDs, which are or are not enriched in methionine residues, and are referred to as MR-PrLD and Non-MR-PrLD, respectively (Table S1). Let us call these sets A and B, respectively. To formalize the network analysis, we applied the methodology described elsewhere [38]. Briefly, we began by defining the sets:

$$O=\left\{all\;the\;GO\;terms\;of\;human\;proteins\right\},\;P=A\;\cup^{\;}B.$$

Next, we defined the mapping *f* as follows:

f:P→𝒫(O)p ↦f(p)={GO terms annotated to protein p},

where 𝒫(*O*) is the powerset of *O*, that is, the set formed by all the subsets of *O*. In this framework, we are in condition to define an endorelation over the set *P*. Thus, we will say that $p_{i}$ and $p_{j}$ are related ($p_{i}$~$p_{j}$), if and only if $p_{i}$ and $p_{j}$ share at least 25% of their *GO* terms. In other words, if their Jaccard similarity index is equal or greater than 0.25:

$$J\left(p_{i},p_{j}\right)=\frac{\left|f\left(p_{i}\right)\cap^{\;}f\left(p_{j}\right)\right|}{\left|f\left(p_{i}\right)\cup^{\;}f\left(p_{j}\right)\right|}\;\geq 0.25.$$

A network is a mathematical graph *G(V,E)*, consisting of a set of nodes or vertices, *V,* and a set of edges, *E*, where its elements are unordered pairs of distinct vertices. In our case, we built and analyzed the following graph:

$$G=(V=P,\;E=\left\{\left(p_{i},p_{j}\right)\in P\;x\;P\;:\;p_{i}\sim p_{j}\right\}.$$

In our network we have vertices either belonging to the set A or to the set B. When vertices of a type show a trend to be related to others that are like them, we say that the network shows assortative mixing. The opposite extreme occurs when the vertices prefer to associate with others that are of different types; then we say that the network shows disassortative mixing. To quantify the level of assortative mixing, we used the assortativity coefficient described by Newman [39] as implemented in the R package igraph [40].

## 3. Results

### 3.1. A Small but Sizeable Fraction of Human Proteins Do Not Contain Methionine Residues Other Than the Initiation One

**Figure 2:**
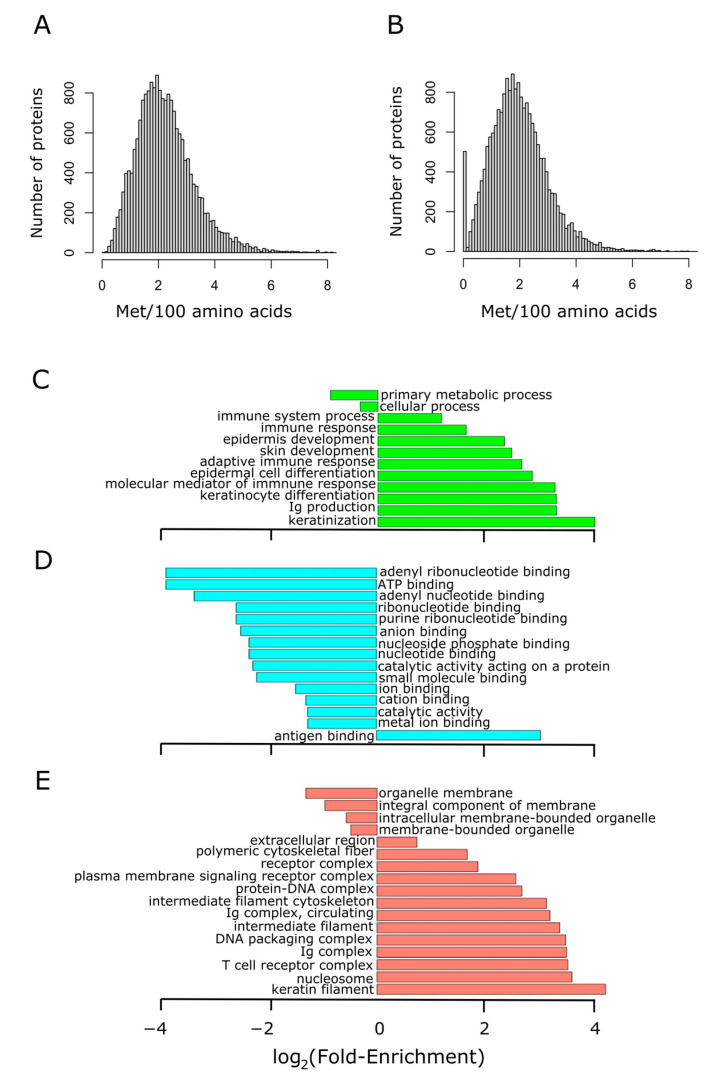
Proteins lacking methionine residues (other than the initiation methionine) are related to immunity. The methionine content distribution of the human proteome is shown in (**A**), where the median was found to be 2.1% (mean 2.2%). The methionine content was recomputed for the human proteome after removing the initiation methionine found at position 1. In this case, the median was located at 1.9% (mean 2%), and a group formed by 500 proteins stands out conspicuously in the distribution (**B**). This group of non-methionine-containing proteins was subjected to GO term enrichment analysis in the three ontologies separately: biological process (**C**), molecular function (**D**), and cellular component (**E**). The abscissa axis shows the binary logarithm of the fold-enrichment, computed as described in Section 2.

We started by addressing the variability in methionine content within the human proteome. Figure 2A shows the histogram of methionine abundance in 19,636 unique proteins of more than 100 residues long. Methionine content showed a normal distribution, slightly skewed to the right. Thus, while methionine had an average occurrence of 2.2%, the spliceosomal protein U1 small nuclear ribonucleoprotein C (the protein exhibiting the highest relative frequency of methionine) reached up to 13.8%. Interestingly, once the initiation methionine was disregarded, a group of around 500 proteins without methionine in their sequences became conspicuous (Figure 2B). This group of proteins, with sizes ranging from 100 to 793 residues in length, was subjected to GO term enrichment analysis. As it can be observed in Figure 2C–E, this group of proteins lacking methionine was related to immunity and skin development. A complete list of these proteins and some of their features (sequence, size, abundance, etc.) can be found in Table S2. 

### 3.2. Aggregation Versus Dispersion of Methionine Residues through the Primary Structure of Human Proteins

**Figure 3:**
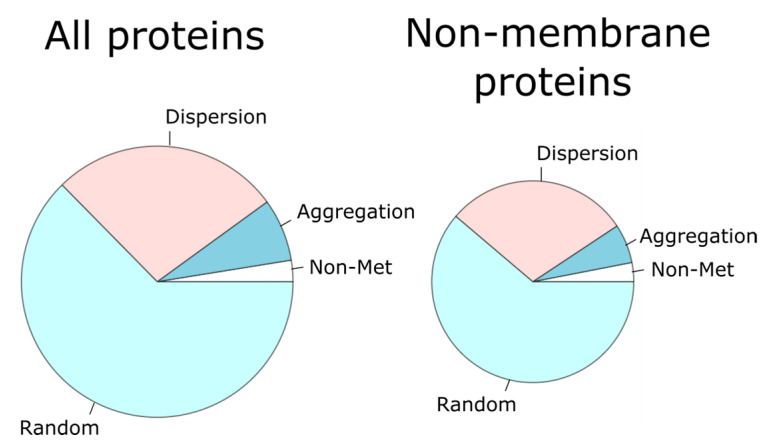
For each protein, the observed distribution of methionine residues along the linear sequence was contrasted with that expected when these residues were randomly distributed. In this way, each protein was sorted into the aggregation, dispersion, or random category. The pie chart on the left represents the percentage of all the proteins in each category. The pie chart on the right gives these proportions for only non-membrane proteins.

For each protein, we investigated the spatial distribution of its methionine residues through the primary structure. To this end we computed a statistic, *q*, that assesses the deviation from the expected values for a purely stochastic process (see the Section 2 for a detailed description). Briefly, the closer *q* is to 0, the greater the dispersion of the methionine residues along the protein sequence. On the contrary, the larger *q* is, the greater the tendency of methionine residues to cluster into patches. In this way, the statistic *q* was computed for each single protein and compared to its own empirical null distribution (see Figure 1 for an example of the methodological procedure), which allowed us to sort out each protein into one of three categories: aggregation, dispersion, and random (Figure 3). For this purpose, when the *q* value computed for a given protein was significantly (*p*-value < 10^−4^) high or low, according to its own empirical distribution, the protein was labeled as ‘aggregation’ or ‘dispersion,’ respectively. Otherwise, the protein was considered to belong to the random group.

Since methionine frequencies in membrane proteins are higher than in non-membrane proteins [41,42], we subdivided our dataset into membrane and non-membrane proteins, observing that proteins classified as ‘aggregation’ were enriched within the membrane protein subset with respect to the non-membrane protein group (*p*-value = 2.2 × 10^−16^, Fisher’s exact test). Subsequently, we filtered out membrane proteins (25.6%) to restrict our analyses to non-membrane proteins (74.4%).

### 3.3. Proteins from the Aggregation Group Tend to Be Met-Enriched and Larger Than Average

**Figure 4:**
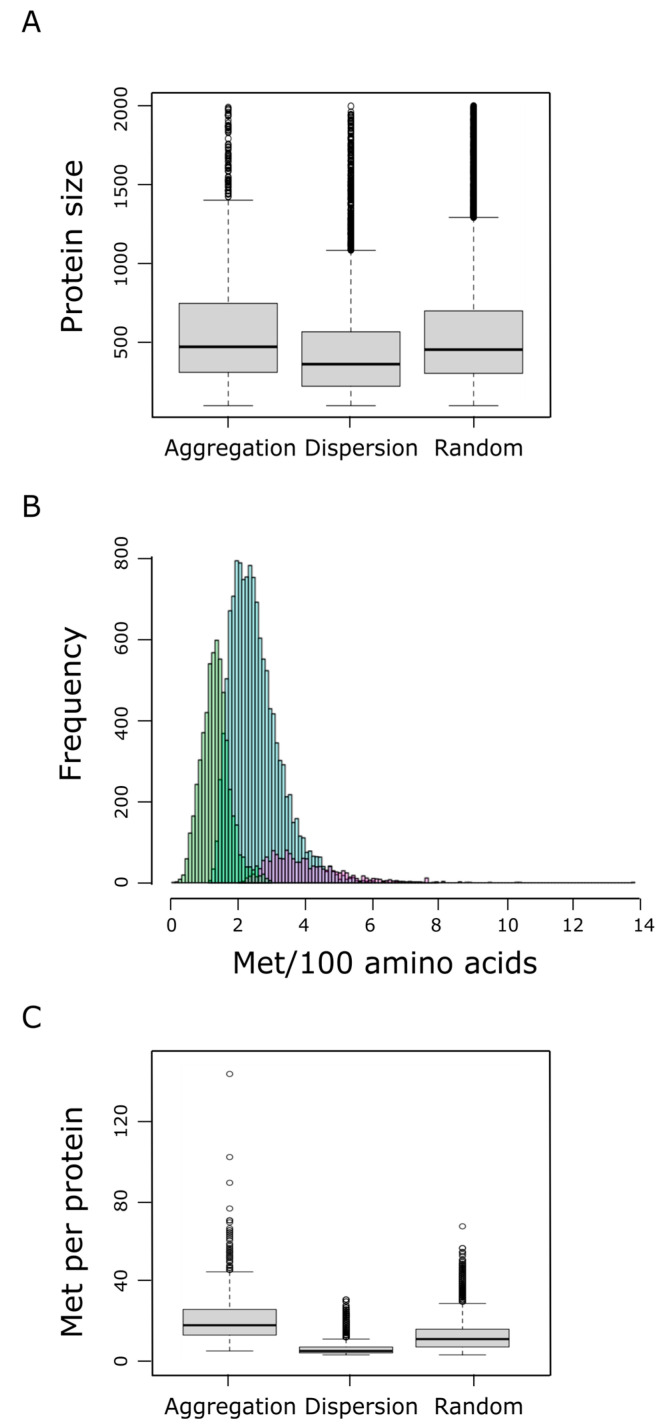
Protein size distributions within the aggregation, dispersion, and random groups are shown in (**A**). The percentage of methionine content in the human proteome, which ranges from 0 to near 14%, was assessed. The absolute frequency of the different methionine contents is shown in (**B**), where differential trends for the dispersion (green), random (blue), and aggregation (purple) groups can be distinguished. In (**C**) the distribution of the absolute methionine frequency for each of these three groups is shown.

We next examined the protein size (number of residues) and the relative frequency of methionine within each protein group. We found that proteins belonging to the aggregation group tend to be larger than proteins from the dispersion category (*p*-value < 2 × 10^−9^, pairwise Wilcoxon rank sum test), and marginally larger than proteins belonging to the random category (Figure 4A). In addition to their propensity to be longer, proteins from the aggregation group also exhibited a higher relative frequency of methionines than random or dispersion proteins (Figure 4B), leading to a highly significant larger number of methionine residues per protein within the aggregation group, when compared to the dispersion (*p*-value < 10^−192^, pairwise Wilcoxon rank sum test) or the random category (*p*-value = 4 × 10^−192^, pairwise Wilcoxon rank sum test) (Figure 4C). At this point, it is important to remember that a protein is labeled as ‘aggregation’ when it forms patches of methionine residues that cannot be explained by chance when a protein of the same length and the same absolute frequency of methionine is considered (Figure 1). Therefore, neither the protein size nor the number of methionine residues can be claimed as direct causal determinants of the tendency to form these methionine patches.

### 3.4. Screening the Aggregation Group for MR-PrLD-Containing Proteins

The human proteome was surveyed in search of proteins that may participate in the regulation of LLPS by the redox status of the protein. To this end, we focused our attention on MR-PrLDs similar to those present in the yeast ataxin-2 and the human TDP-43 proteins [17]. Since direct sequence comparison via sequence alignment was not a feasible approach, we resorted to a definition of MR-PrLD that would be easy to handle computationally. For this purpose, we defined a MR-PrLD as any sequence stretch within a protein from the ‘aggregation’ group that could be classified as an LC region. In addition, this region should contain at least five methionine residues and, with a probability greater than 0.9, would be predicted as a prion-like domain. In this way, we identified 51 proteins accounting for 78 MR-PrLDs (Table S3). 

**Figure 5:**
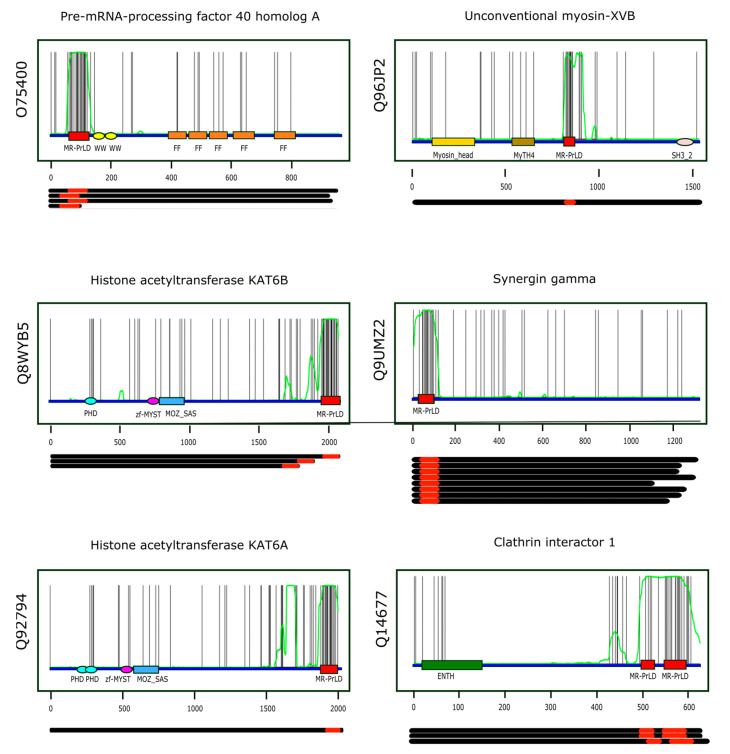
Multi-modular architecture of MR-PrLD-containing proteins. For the six top-ranking proteins in the MR-PrLD census (the Uniprot identifier is indicated vertically to the left of each plot, and the protein name horizontally at the top of each plot), the positions of the methionine residues along the primary structure are represented by black vertical lines. Along the protein sequence (blue segment), we have marked the Pfam domains detected in each protein using colored rectangles and ellipses. The MR-PrLDs are represented as red boxes. The green curve represents the probability of the residue at that position being part of a PrLD according to PLAAC [33], an algorithm based on hidden-Markov models. Each plot has been done using the canonical form of the protein being analyzed. Below each plot, the length of the different isoforms known to be produced by alternative splicing are represented as black segments, and their MR-PrLDs as red rectangles.

Each one of these MR-PrLDs was scored according to the unlikeliness of observing so many methionine residues clustered together by chance. For this scoring, the length (*n*) and the number of methionine residues (*x*) was counted for each MR-PrLD. On the other hand, the relative frequency of methionine in the whole protein (*p*) was also computed. In this way, a *p*-value could be calculated as follows:

(2) p−Value=P[X≥x],

assuming that the random variable *X* (number of methionines found along the sequence stretch) follows a binomial distribution of parameters *n* and *p*, X~Bin(n,p). Table 1 presents information on the first six proteins when ranked according to increasing *p*-values, while Figure 5 shows the multi-modular architecture of these proteins. A comprehensive list of the Pfam domains accompanying MR-PrLDs can be found in Table S4.

### 3.5. Proteins Containing MR-PrLDs Are Extraordinarily Compact

**Figure 6:**
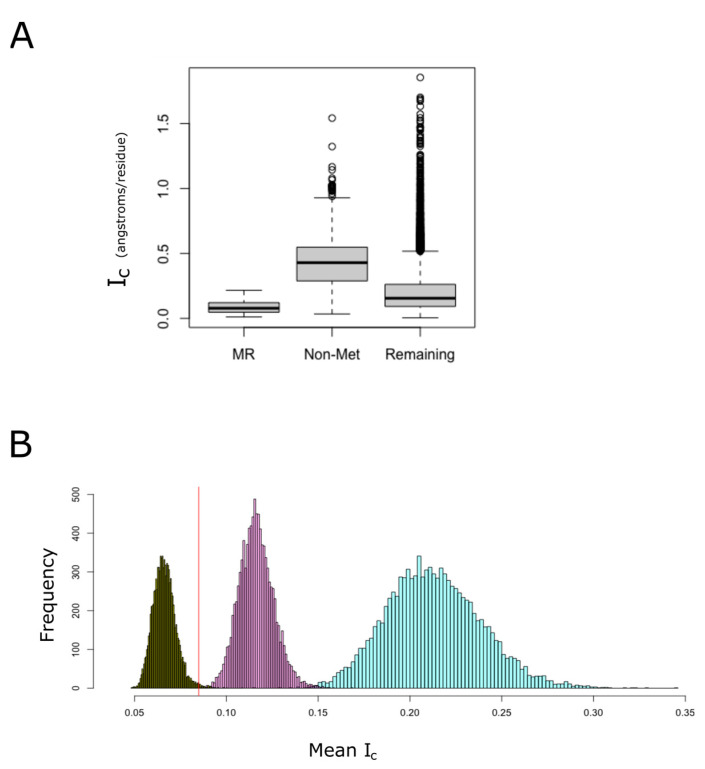
MR-PrLD-containing proteins are more compact than most proteins. Among non-membrane proteins, we distinguished three categories: (i) proteins belonging to the MR-PrLD set (de-noted as MR in the figure), (ii) proteins lacking methionine, other than the one for initiation, in their sequences (denoted as Non-Met in the figure), and (iii) the remaining proteins. For each of these categories, the index of compactness, *I_c_*, is plotted using boxplots (**A**). In (**B**), the empirical null distributions of $I_{c}$ mean for 49 proteins randomly sampled from the non-membrane human proteome are shown. Random sampling was carried out in three different ways: (i) without restriction (turquoise color), (ii) matching the protein size with that of the MR-PrLD group (purple color), and (iii) matching the methionine content (dark green color). The computed $I_{c}$ mean value for the MR-PrLD set was 0.085 angstrom per residue, and it is indicated by a vertical red line in the plot.

LCRs and prion-like domains tend to be intrinsically disordered [17]. These protein regions fail to form a unique predominantly stable tertiary structure, which favors less compact conformations [43]. However, despite that MR-PrLDs are expected to be intrinsically disordered, we noted that proteins from the MR-PrLD set are, on average, more compact than most non-membrane proteins from the human proteome, and much more compact than proteins from the group of proteins lacking methionine (Figure 6A). To quantify the probability that this observation could be explained by chance due to sampling effects, we built empirical distributions of the $I_{c}$ by randomly sampling proteins from the non-membrane proteome and computing their means (Figure 6B). When the null distribution was built by sampling proteins from the non-membrane proteome without any further restriction, we observed a normal distribution, with a mean of 0.213 and a standard deviation of 0.026. The mean value for the group of proteins containing MR-PrLDs was significantly less at 0.085 (*p*-value < 0.0001). Even when we controlled for the effect of protein size on the $I_{c}$, we concluded that MR-PrLD-containing proteins are more compact than expected by chance in proteins of the same size (*p*-value = 0.0001). Conversely, when we controlled for methionine content instead of protein size, we observed that proteins from the MR-PrLD set were less compact than expected for proteins with such a high methionine abundance (*p*-value = 0.008).

### 3.6. GO Analysis of MR-PrLD-Containing Proteins 

To gain insights into the biological processes in which these MR proteins may be involved, we next carried out a GO term enrichment analysis using the MR-PrLD proteins as a target set, against the whole human reference proteome as a background set. In this way, we found a statistically significant enrichment in GO terms related to RNA and DNA associated processes, including transcription and chromatin organization and remodeling, as well as terms linked to developmental processes, among others. A full list of enriched terms and their statistical details can be found at Table S1. At first glance, this result might seem very similar to that reported by Iglesias et al. for a group of computationally identified human prion-like proteins for which GO terms pointing to RNA and DNA processes were at the top of their list [37]. Since among these proteins, methionine was not a particularly abundant amino acid, this group will be referred to as Non-MR-PrLD. 

**Figure 7:**
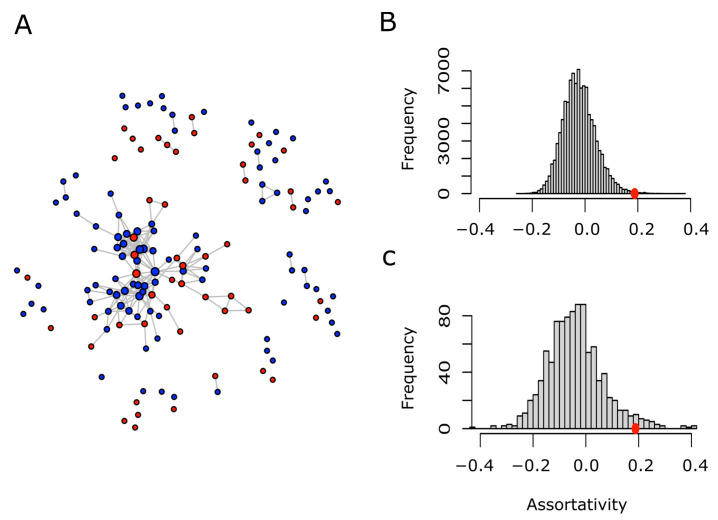
Network of human PrLD proteins and assortativity analysis. Two given proteins (nodes) are connected (linked by an edge) if they share at least 25% of their GO terms. Proteins belonging to the MR-PrLD set are shown in red, while those from the Non-Met-PrLD set are shown in blue (**A**). Retaining the network topology and the proportion of red and blue nodes, each node was randomly relabeled as red or blue, then the assortativity coefficient of the relabeled network was computed. This process was repeated 105 times, and the assortativity distribution was plotted (**B**). On the other hand, the human proteome was randomly sampled to obtain as many proteins as nodes present in A. Using the same binary relationship (two proteins are connected if they share at least 25% of their GO terms), a network was built. Afterwards, each node was randomly labeled as red or blue, keeping the proportion shown in A. This process was repeated 1000 times and, in each case, the assortativity coefficient was computed. The distribution of the assortativity coefficient computed in this way was plotted (**C**). The red circles in B and C point to the assortativity coefficient value (0.188) computed for the actual network shown in A.

The considerable overlap in GO terms present in both sets (MR-PrLD and Non-MR-PrLD) was not surprising, since both were formed for modular prion-like containing proteins. Nevertheless, we were not so much interested in the similarities as in the differences. Thus, to search for the particularities, if any, of the MR-PrLD-containing proteins, we designed a network analysis. Briefly, each PrLD protein was considered as a vertex or node, and was labeled as red (MR-PrLD) or blue (Non-MR-PrLD). Two nodes were related to each other if they shared at least a 25% of the GO terms describing both proteins (nodes). Once we introduced such a binary relation, we were able to take full advantage of the network theory to answer our question of interest: are MR-PrLD nodes more often related among themselves than they are related to Non-MR-PrLD nodes? A straightforward, quantitative, and visual way to answer this question is by drawing the network and computing its assortativity. In general, for a network formed by two types of nodes, the assortativity coefficient can range between −1, when the network is completely disassortative (every edge connects two nodes of different types) and 1, when there is perfect assortative mixing (every edge connects two nodes of the same type). When the link between two nodes is not influenced by the type of the nodes, the assortativity coefficient takes values close to zero. Using this approach, we built a network of the human PrLD-containing proteins (Figure 7A), and computed its assortativity coefficient, which turned out to be 0.188. Since this value is greater than 0, we concluded that there was a positive assortativity. Nevertheless, to assess the statistical strength of such a conclusion, we performed two different types of controls based on either the random relabeling of nodes or the random sampling of nodes (Figure 7B,C). In both cases, we could conclude that the positive assortativity observed for the human PrLD network was statistically significant (*p*-values 0.003 and 0.034, respectively). Details regarding the GO terms specific for MR-PrLD or Non-MR-PrLD can be obtained in Table S1.

## 4. Discussion

The aim of the current study was to elaborate a census of human proteins that may be involved in LLPS processes regulated by the redox status of the cell. To guide the search, we took as a model the yeast ataxi-2 and the human TDP-43 proteins. These two proteins exhibit a completely unrelated primary structure. Nevertheless, both proteins present in their amino acid sequence a region of low complexity, rich in methionine residues, which can be identified as a prion-like domain [17]. On the other hand, these MR-PrLDs have been experimentally proved to be involved in the formation of droplets in a redox-regulated fashion. Furthermore, this redox regulation seems to take place via reversible methionine oxidation [23,24]. In this context, we selected four criteria that had to be satisfied simultaneously for a given protein to be considered as a MR-PrLD-containing protein: (i) the distribution of the methionine residues throughout the primary structure should show an aggregation trend; (ii) an LCR containing methionine residues should be detected in the protein sequence, (iii) these LCRs must coincide with sequence stretches identified as PrLDs, and (iv) the sequence stretch must contain at least 5 methionine residues.

In relation to the first of these criteria, it is a well-known fact that the average methionine content of human proteins is around 2%. Nevertheless, the amino acid composition of proteins depends on their size [44]. Since for structured proteins, the surface-to-volume ratio decreases with increasing protein size and, on the other hand, polar amino acids tend to be located on the surface of proteins, while apolar amino acids are buried within the protein core, the influence of protein size on amino acid composition is not surprising. However, a significant fraction of amino acid residues from a proteome is found at unstructured regions [45], where the surface-to-volume rule might not hold. Furthermore, even for structured proteins, methionine can be found both exposed at the surface and buried within the protein interior [46,47]. In short, very little is known regarding the variability of methionine content in human proteins and its functional significance. Thus, we started by addressing how methionine residues tend to be distributed through the primary structure of proteins.

We analyzed the distribution of 240,507 methionine residues among 19,636 human proteins, accounting for 11,302,527 residues. Thus, methionine content in human proteins averaged, as expected, around 2%. More interestingly, about 500 proteins, with sizes ranging from 100 to 793 residues, stood out by not presenting methionine in their sequence, apart from the initiation methionine. Gene ontology terms enrichment analysis of this group of proteins revealed that this set was enriched with proteins related to the skin and immunity (Figure 2). As intriguing as this observation is, since our original goal was something else, we did not further investigate the potential reasons why proteins related to epidermis development and immunity avoid methionine residues in their sequences. On the other end of the distribution, there was a group of nearly 200 proteins with an abundance of methionine above the 5% (mean plus three standard deviations). Nevertheless, our main interest was more focused on how methionine residues were distributed along the primary structure of proteins than in their abundance. Therefore, we sorted all the non-membrane human proteins into three categories (random, dispersion, and aggregation) according to probabilistic criteria using random models. Proteins belonging to the aggregation group (6.3%) were the least numerous of the three categories (Figure 3).

At this point, we were able to address the question: What is, if any, the relationship between methionine abundance and the trend of this amino acid to aggregate or disperse? In this respect, we observed that large and methionine-rich proteins were significantly overrepresented in the aggregation group (Figure 4). It should be noted that this result could not be anticipated. Indeed, there is no obvious reason why a protein that has few methionines should not cluster them together in a short stretch of its sequence. On the other hand, a protein with a high number of methionine residues might, a priori, scatter them throughout the whole sequence. Thus, our comprehensive analysis regarding the dependence of both factors, protein size and methionine usage, on the aggregation-dispersion trend of methionine residues (Figure 4, Figures S1 and S2) provides an answer to a previously unresolved question. Thus, the current results allow us to conclude that proteins that exhibit methionine aggregation are larger than average, with larger relative methionine frequencies than average human proteins.

Among this set of proteins that exhibited a trend to cluster their methionine residues together, we searched for domains that may be potentially involved in the redox regulation of LLPS processes. To guide this search, the properties of the methionine-rich domains found into the yeast ataxin-2 and the human TDP-43 proteins were considered. These domains were taken as a reference model because it has been shown experimentally that they are involved in the regulation, via reversible methionine oxidation, of the assembly/disassembly of biomolecular condensates (reviewed in [17]). Consequently, we defined an MR-PrLD as a low complexity sequence stretch, rich in methionine, and predicted as prion-like domain. In this way, we detected 78 MR-PrLDs in 51 different proteins. Each of these domains were ordered according to a *p*-value, and the top (more significant) 6 are shown into Table 1.

Two remarkable features of those proteins that have MR-PrLDs are their modular architecture (Figure 5) and their above-average compactness. The first of these properties, that is, the modular nature of these proteins, is not surprising, since most proteins able to undergo phase separation usually present a modular design [17]. More surprising, at first glance, might be the high degree of compactness observed within the MR-PrLDs-containing proteins (Figure 6). That is because LCRs are expected to be disordered structures and, in contrast, proteins presenting a high LCR content would be expected to be less compact than well-structured proteins [43]. However, it should be considered that the LCRs from MR-PrLDs differ substantially in their composition from more standard LCRs. In the latter, polar amino acids such as glutamine, serine, proline and glycine are often over-represented, while large and/or non-polar amino acids, including methionine, are under-represented [4,48]. Therefore, MR-PrLDs are much more hydrophobic than conventional PrLDs. This high methionine content, and the subsequent hydrophobicity of the domain, might be behind the high mean compactness observed for the set of proteins containing MR-PrLDs, as suggested by our sampling experiments (Figure 6B).

The yeast poly(A)-binding protein, Pab1, is a modular protein containing a MR-PrLD [17]. Interestingly, Riback et al. showed that the unstructured MR-LCR of this protein exhibited a hydrophobicity-dependent compaction. Thus, reducing hydrophobicity with methionine to alanine substitutions increased the domain’s radius of gyration (Rg). Conversely, increasing hydrophobicity with methionine to isoleucine substitutions decreased Rg. These authors point out that the intramolecular interactions that cause this MR-PrLD compaction may also contribute to intermolecular interactions that influence phase separation of the Pab1 protein [49]. Therefore, it seems reasonable to speculate that the human proteins containing MR-PrLDs described in the current work, which exhibit an unusual compaction, may regulate their compactness via the reversible oxidation of methionine to methionine sulfoxide (MetO). It is a well-known fact that the oxidation of methionine contributes polarity to an otherwise apolar side chain [25]. Thus, the hydrophobicity index decreases from 0.738 for Met to 0.238 for MetO, very similar to that of glutamine (0.251) [50], an amino acid often found in standard non-MR-PrLDs [51].

In the context of the current investigation, human proteins containing prion-like domains have been classified as either MR or non-MR. Both groups share several features, including a modular design suitable for its participation in processes of the regulation of the flow of genetic information (Table S1). However, we were much more interested in emphasizing their differences, with the aim of getting an insight into those cellular processes involving LLPS that might be redox regulated. For this purpose, we carried out network analyses using GO terms to define relationships between proteins, addressing the assortativity between both groups of PrLD-containing proteins. The results of such analyses allow us to conclude that despite the similarities between the MR and non-MR sets, MR-PrLD proteins selectively share enough GO terms with each other to allow a clear discrimination, with respect to the non-MR-PrLD group (Figure 7 and Table S1), suggesting that high methionine content and high compaction might be suitable for specific functions involving specific prion-like domains. A relevant functional role for these MR-PrLDs is further supported for the observation that these domains appear to be conserved throughout their evolution (Figures S3 and S5).

## 5. Conclusions

Until now, only a reduced number of proteins containing prion-like domains enriched with methionine have been described in the literature. The experimental characterization of these few proteins has revealed a hitherto new biological function for the reversible oxidation of methionine. Concretely, the interconversion of methionine and methionine sulfoxide can function to regulate liquid–liquid phase separation and the subsequent assembly/disassembly of supramolecular structures in response to redox stimuli. 

Although so far only a few proteins exemplify this novel function of methionine residues, we know, as noted by François Jacob, that evolution always repeatedly reuses successful designs, in slightly modified variations [52]. Thus, prompted by this reasoning, we have performed a proteome-wide survey to search for human proteins containing methionine-rich prion-like domains (MR-PrLDs). In this way, we have found 51 different proteins accounting for 78 MR-PrLDs, which have been ranked according to the unlikeliness of observing them by chance. The subsequent computational characterization of these proteins revealed several properties that they have in common: (i) the MR-PrLDs present in these proteins tend to be evolutionary conserved, as suggested by the fact that they were detected in orthologous proteins from other mammalian species. (ii) Proteins containing MR-PrLDs were significantly larger than average, and (iii) they exhibited modular architectures; (iv) remarkably, these proteins showed an unusually high degree of compactness, most probably due to the high local concentration of methionine residues.

In summary, this study provides a census of human MR-PrLD-containing proteins that share a series of properties that make them particularly suitable to promote, in a redox regulated fashion, protein aggregation and liquid–liquid phase separation. We hope that the current work will inspire future experimental research to further explore and confirm the proposed functional role of these methionine-rich motifs.

## Figures and Tables

**Table 1:** Top-ranking MR-PrLDs according to the *p*-value criterion. The six top-ranking MR-PrLDs containing proteins were: (i) pre-mRNA-processing factor 40 homolog A (O74500); (ii) histone acetyltransferase KAT6B (Q8WYB5); (iii) histone acetyltransferase KAT6A (Q92794); (iv) unconventional myosin-XVB (Q96JP2); (v) synergin gamma (Q9UMZ2); and (vi) clathrin interactor 1 (Q14677).

ID	Start	End	Length	x	*p*-Value	MR-PrLD
O75400	65	121	57	19	1.79 × 10^−13^	PMGMHPMGQRANMPPVPHGMMPQMMPPMGGPPMGQMPGMMSSVMPGMMMSHMSQASM
Q8WYB5	1961	2068	108	21	4.53 × 10^−13^	MQRGMNMSVNLMPAPAYNVNSVNMNMNTLNAMNGYSMSQPMMNSGYHSNHGYMNQTPQYPMQMQMGMMGTQPYAQQPMQTPPHGNMMYTAPGHHGYMNTGMSKQSLNG
Q92794	1894	1977	84	18	5.74 × 10^−12^	QRGMNMGVNLMPTPAYNVNSMNMNTLNAMNSYRMTQPMMNSSYHSNPAYMNQTAQYPMQMQMGMMGSQAYTQQPMQPNPHGNMM
Q96JP2	820	847	28	8	6.25 × 10^−9^	PMVYPGMIQMPAYQPGMVPAPMPMMPAM
Q9UMZ2	37	94	58	12	9.29 × 10^−9^	PPQAGLMPMQQQGFPMVSVMQPNMQGIMGMNYSSQMSQGPIAMQAGIPMGPMPAAGMP
Q14677	549	591	43	14	1.72 × 10^−8^	MPMSMPNVMTGTMGMAPLGNTPMMNQSMMGMNMNIGMSAAGMG

## Data Availability

Data are contained within the article and Supplementary Materials. The R scripts written ad hoc for this study can be found at [34].

## Supplementary Materials

The following supporting information can be downloaded at: https://www.mdpi.com/article/10.3390/antiox11071289/s1, Figure S1: Effect of protein size on the null distribution of the *q*-statistic; Figure S2: Effect of methionine abundance on the null distribution of the *q*-statistic; Figure S3: Evolutionary conservation of MR-PrLDs in mammalian species; Table S1: GO terms enrichment analysis of MR-PrLD- and Non-MR-PrLD-containing human proteins; Table S2: Identification and features of human proteins lacking methionine residues; Table S3: Identification of 78 MR-PrLDs present in 51 different human proteins; Table S4: List of the Pfam domains accompanying MR-PrLDs; Table S5: Details regarding MR-PrLDs found in sequences from different mammalian species.

## References

[1] Collins F.S., Lander E.S., Rogers J. (2004). International Human Genome Sequencing Consortium, Finishing the euchromatic sequence of the human genome. Nature.

[2] Gibbs R.A. (2020). The Human Genome Project changed everything. Nat. Rev. Genet..

[3] Kato M., Zhou X., McKnight S.L. (2022). How do protein domains of low sequence complexity work?. RNA.

[4] Haerty W., Golding G.B. (2010). Low-complexity sequences and single amino acid repeats: Not just “junk” peptide sequences. Genome.

[5] Ntountoumi C., Vlastaridis P., Mossialos D., Stathopoulos C., Iliopoulos I., Promponas V., Oliver S.G., Amoutzias G.D. (2019). Low complexity regions in the proteins of prokaryotes perform important functional roles and are highly conserved. Nucleic Acids Res..

[6] Ellegren H. (2004). Microsatellites: Simple sequences with complex evolution. Nat. Rev. Genet..

[7] Ventura S., Zurdo J., Narayanan S., Parreño M., Mangues R., Reif B., Chiti F., Giannoni E., Dobson C.M., Aviles F.X. (2004). Short amino acid stretches can mediate amyloid formation in globular proteins: The Src homology 3 (SH3) case. Proc. Natl. Acad. Sci. USA.

[8] Gatchel J., Zoghbi H. (2005). Diseases of Unstable Repeat Expansion: Mechanisms and Common Principles. Nat. Rev. Genet..

[9] Shin J.S., Chung K.W., Cho S.Y., Yun J., Hwang S.J., Kang S.H., Cho E.M., Kim S.-M., Choi B.-O. (2008). NEFL Pro22Arg mutation in Charcot-Marie-Tooth disease type 1. J. Hum. Genet..

[10] Ryan V., Dignon G.L., Zerze G.H., Chabata C.V., Silva R., Conicella A.E., Amaya J., Burke K.A., Mittal J., Fawzi N.L. (2018). Mechanistic View of hnRNPA2 Low-Complexity Domain Structure, Interactions, and Phase Separation Altered by Mutation and Arginine Methylation. Mol. Cell.

[11] Qi X., Pang Q., Wang J., Zhao Z., Wang O., Xu L., Mao J., Jiang Y., Li M., Xing X. (2017). Familial Early-Onset Paget’s Disease of Bone Associated with a Novel hnRNPA2B1 Mutation. Calcif. Tissue Res..

[12] Erro M.E., Zelaya M.V., Mendioroz M., Larumbe R., Ortega-Cubero S., Lanciego J.L., Lladó A., Cabada T., Tuñón T., García-Bragado F. (2019). Globular glial tauopathy caused by MAPT P301T mutation: Clinical and neuropathological findings. J. Neurol..

[13] Goedert M., Jakes R. (2005). Mutations causing neurodegenerative tauopathies. Biochim. Biophys. Acta (BBA)-Mol. Basis Dis..

[14] Rizzu P., Joosse M., Ravid R., Hoogeveen A., Kamphorst W., Van Swieten J.C., Willemsen R., Heutink P. (2000). Mutation-dependent aggregation of tau protein and its selective depletion from the soluble fraction in brain of P301L FTDP-17 patients. Hum. Mol. Genet..

[15] Zhou X., Lin Y., Kato M., Mori E., Liszczak G., Sutherland L., Sysoev V.O., Murray D.T., Tycko R., McKnight S.L. (2021). Transiently structured head domains control intermediate filament assembly. Proc. Natl. Acad. Sci. USA.

[16] Harrison P.M. (2018). Compositionally Biased Dark Matter in the Protein Universe. Proteomics.

[17] Aledo J.C. (2021). The Role of Methionine Residues in the Regulation of Liquid-Liquid Phase Separation. Biomolecules.

[18] Kato M., Han T.W., Xie S., Shi K., Du X., Wu L.C., Mirzaei H., Goldsmith E.J., Longgood J., Pei J. (2012). Cell-free Formation of RNA Granules: Low Complexity Sequence Domains Form Dynamic Fibers within Hydrogels. Cell.

[19] Xiang S., Kato M., Wu L., Lin Y., Ding M., Zhang Y., Yu Y., McKnight S. (2015). The LC Domain of hnRNAPA2 Adopts Similar Conformations in Hydrogel Polymers, Liquid-like Droplets and Nuclei. Cell.

[20] Yang Y.-S., Kato M., Wu X., Litsios A., Sutter B.M., Wang Y., Hsu C.-H., Wood N.E., Lemoff A., Mirzaei H. (2019). Yeast Ataxin-2 Forms an Intracellular Condensate Required for the Inhibition of TORC1 Signaling during Respiratory Growth. Cell.

[21] Franzmann T.M., Alberti S. (2019). Prion-like low-complexity sequences: Key regulators of protein solubility and phase behavior. J. Biol. Chem..

[22] Wang B., Zhang L., Dai T., Qin Z., Lu H., Zhang L., Zhou F. (2021). Liquid–liquid phase separation in human health and diseases. Signal Transduct. Target. Ther..

[23] Kato M., Yang Y.-S., Sutter B.M., Wang Y., McKnight S.L., Tu B.P. (2019). Redox State Controls Phase Separation of the Yeast Ataxin-2 Protein via Reversible Oxidation of Its Methionine-Rich Low-Complexity Domain. Cell.

[24] Lin Y., Zhou X., Kato M., Liu D., Ghaemmaghami S., Tu B.P., McKnight S.L. (2020). Redox-mediated regulation of an evolutionarily conserved cross-β structure formed by the TDP43 low complexity domain. Proc. Natl. Acad. Sci. USA.

[25] Aledo J.C. (2019). Methionine in proteins: The Cinderella of the proteinogenic amino acids. Protein Sci..

[26] Human Proteome UP000005640. https://www.uniprot.org/proteomes/UP000005640.

[27] Blake J.A., Christie K.R., Dolan M.E., Drabkin H.J., Hill D.P., Ni L., Sitnikov D., Burgess S., Buza T., Gresham C. (2015). Gene Ontology Consortium: Going forward. Nucleic Acids Res..

[28] Mi H., Muruganujan A., Casagrande J., Thomas P. (2013). Large-scale gene function analysis with PANTHER Classification System. Nat. Protoc..

[29] Hayes J.J., Castillo O. (2017). A New Approach for Interpreting the Morisita Index of Aggregation through Quadrat Size. ISPRS Int. J. Geo-Inf..

[30] McVinish R., Lester R.J.G. (2020). Measuring aggregation in parasite populations. J. R. Soc. Interface.

[31] Harrison P.M. (2017). fLPS: Fast discovery of compositional biases for the protein universe. BMC Bioinform..

[32] Harrison P.M. (2021). fLPS 2.0: Rapid annotation of compositionally-biased regions in biological sequences. PeerJ.

[33] Lancaster A., Nutter-Upham A., Lindquist S., King O.D. (2014). PLAAC: A web and command-line application to identify proteins with prion-like amino acid composition. Bioinformatics.

[34] R Scripts Accompanying the Current Paper. https://bitbucket.org/jcaledo/mr-prld/src/master/Scripts/MetDistribution.R.

[35] Jumper J., Evans R., Pritzel A., Green T., Figurnov M., Ronneberger O., Tunyasuvunakool K., Bates R., Žídek A., Potapenko A. (2021). Highly accurate protein structure prediction with AlphaFold. Nature.

[36] Varadi M., Anyango S., Deshpande M., Nair S., Natassia C., Yordanova G., Yuan D., Stroe O., Wood G., Laydon A. (2022). AlphaFold Protein Structure Database: Massively expanding the structural coverage of protein-sequence space with high-accuracy models. Nucleic Acids Res..

[37] Iglesias V., Paladin L., Juan-Blanco T., Pallares I., Aloy P., Tosatto S.C.E., Ventura S. (2019). In silico Characterization of Human Prion-Like Proteins: Beyond Neurological Diseases. Front. Physiol..

[38] Aledo J.C., Aledo P. (2020). Susceptibility of Protein Methionine Oxidation in Response to Hydrogen Peroxide Treatment–Ex Vivo Versus In Vitro: A Computational Insight. Antioxidants.

[39] Newman M.E.J. (2003). Mixing patterns in networks. Phys. Rev. E-Stat. Phys. Plasmas Fluids Relat. Interdiscip. Top.

[40] Csardi G., Nepusz T. (2006). The igraph software package for complex network research. Inter. J. Complex Syst..

[41] Gómez-Tamayo J.C., Cordomí A., Olivella M., Mayol E., Fourmy D., Pardo L. (2016). Analysis of the interactions of sulfur-containing amino acids in membrane proteins. Protein Sci..

[42] Mbaye M.N., Hou Q., Basu S., Teheux F., Pucci F., Rooman M. (2019). A comprehensive computational study of amino acid interactions in membrane proteins. Sci. Rep..

[43] Toll-Riera M., Radó-Trilla N., Martys F., Albà M.M. (2011). Role of Low-Complexity Sequences in the Formation of Novel Protein Coding Sequences. Mol. Biol. Evol..

[44] Carugo O. (2008). Amino acid composition and protein dimension. Protein Sci..

[45] Ward J., Sodhi J., McGuffin L., Buxton B., Jones D. (2004). Prediction and Functional Analysis of Native Disorder in Proteins from the Three Kingdoms of Life. J. Mol. Biol..

[46] Lim J.M., Kim G., Levine R.L., Heart N. (2019). Methionine in proteins: It’ s not just for protein initiation anymore. Neurochem. Res..

[47] Janin J., Miller S., Chothia C. (1988). Surface, subunit interfaces and interior of oligomeric proteins. J. Mol. Biol..

[48] Marcotte E., Pellegrini M., Yeates T., Eisenberg D. (1999). A census of protein repeats. J. Mol. Biol..

[49] Riback J.A., Katanski C.D., Kear-Scott J.L., Pilipenko E.V., Rojek A.E., Sosnick T.R., Drummond D.A. (2017). Stress-Triggered Phase Separation Is an Adaptive, Evolutionarily Tuned Response. Cell.

[50] Black S.D., Mould D.R. (1991). Development of hydrophobicity parameters to analyze proteins which bear post- or cotranslational modifications. Anal. Biochem..

[51] Michelitsch M.D., Weissman J.S. (2000). A census of glutamine/asparagine-rich regions: Implications for their conserved function and the prediction of novel prions. Proc. Natl. Acad. Sci. USA.

[52] Jacob F. (1977). Evolution and Tinkering. Science.

