# Supplementary material for: A Census of Human Methionine-Rich Prion-like Domain-Containing Proteins
Source: Antioxidants (Basel). 2022 Jun 29;11(7):1289. doi: 10.3390/antiox11071289 (PMC9312190; doi:10.3390/antiox11071289)
Supplement: Supplementary file 1 [file antioxidants-11-01289-s001.zip › Supplementary_Material_Census.pdf]

Supplementary Material

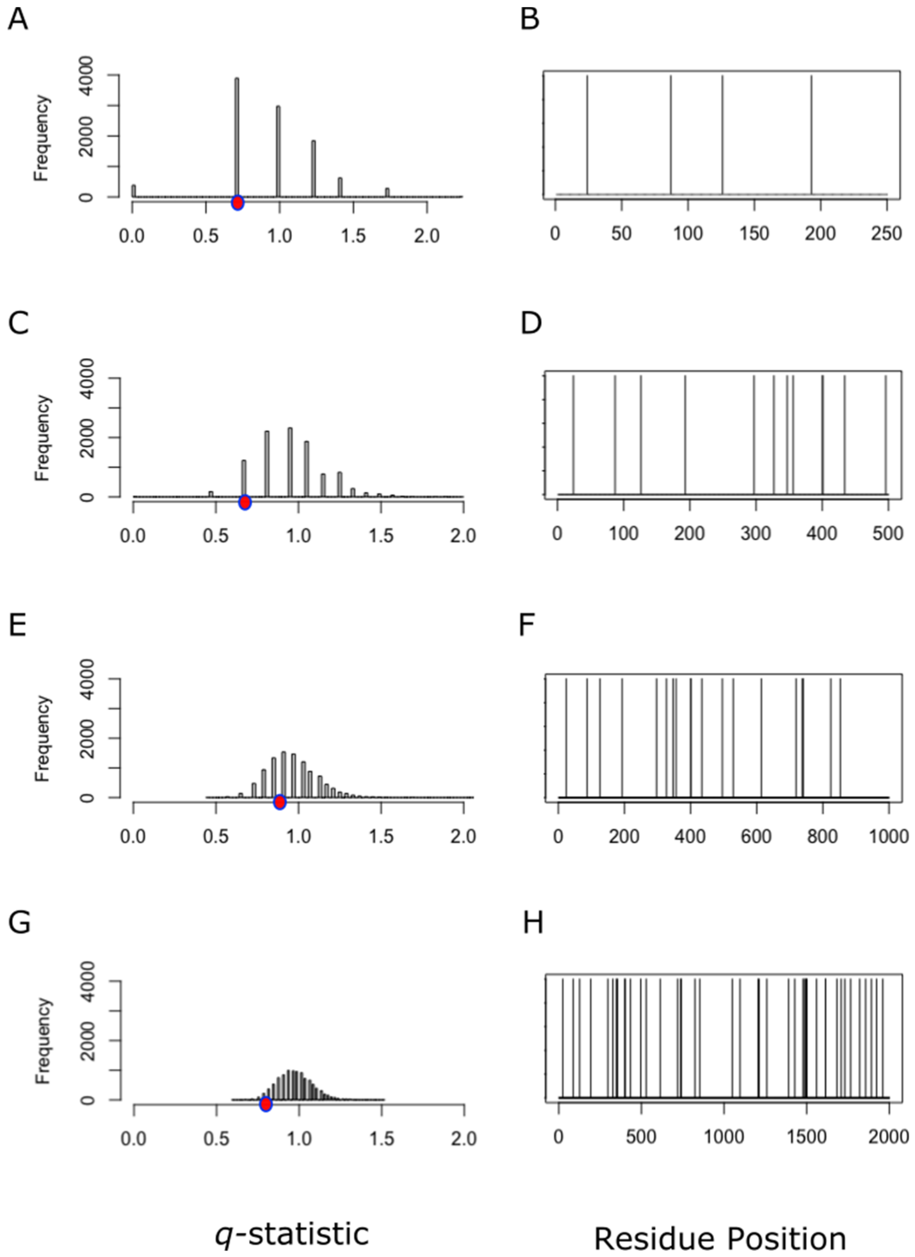

Figure S1. Effect of protein size on the null distribution of the q-statistic. At a fixed frequency of methionine (2 %), four different protein sizes were considered: 250- (A and B), 500- (C and D), 1000- (E and F) and 2000-residue long (G and H) proteins. In all the cases methionine residues were randomly distributed across the lineal protein sequence in 10,000 simulations for each protein size, and the corresponding coefficient of variation, *q*, was computed and its distribution plotted (A, C, E and G). One out the 10,000 simulations was randomly chosen to show the disposition of methionine residues across the sequence (B, D, F and H) that is compatible with the corresponding *q*-value indicated by the red circle in A, C, E and G, respectively.

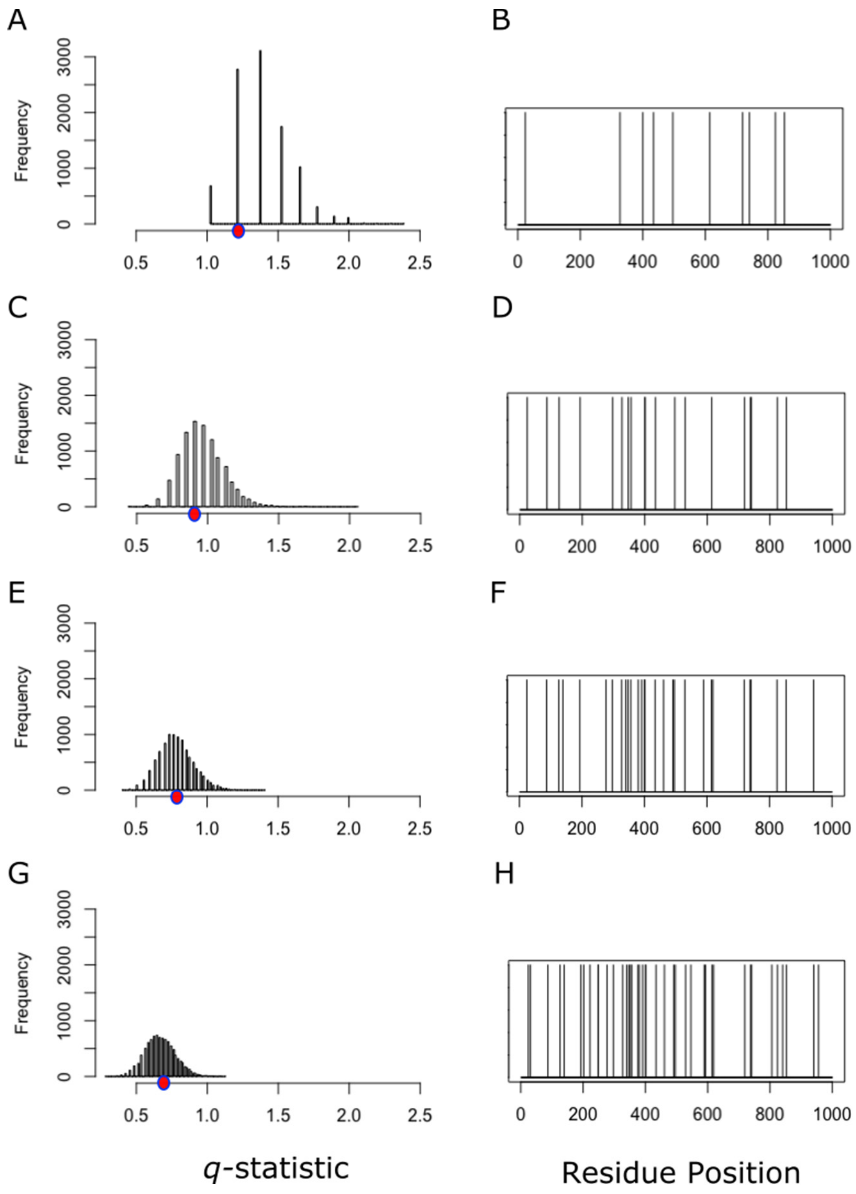

Figure S2. Effect of methionine abundance on the null distribution of the *q*-statistic. A 1000-residue long protein was considered at four different methionine frequencies: 1 % (A and B), 2 % (C and D), 3 % (E and F) and 4 % (G and H). In all the cases methionine residues were randomly distributed across the lineal protein sequence in 10,000 simulations for each methionine frequency, and the corresponding coefficient of variation, *q*, was computed and its distribution plotted (A, C, E and G). One out the 10,000 simulations was randomly chosen to show the disposition of methionine residues across the sequence (B, D, F and H) that is compatible with the corresponding *q*-value indicated by the red circle in A, C, E and G, respectively.

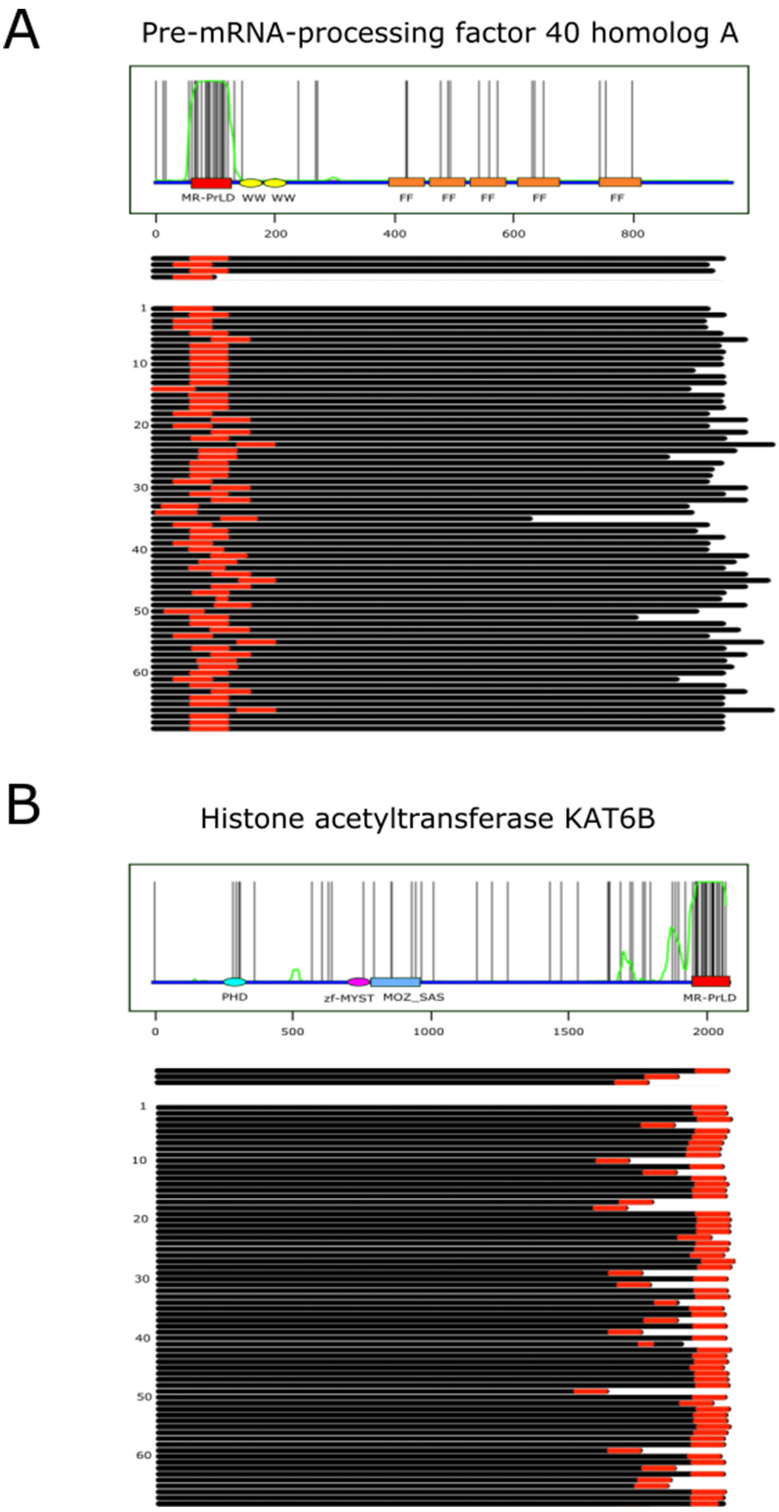

Figure S3. Evolutionary conservation of MR-PrLDs in mammalian species. Among the top scoring proteins from the census presented in this study, two of them were selected to represent proteins with the MR-PrLD at the N-terminal (Pre-mRNA-processing factor 40 homolog A, O75400) and at the C-terminal (Histone acetyltransferase KAT6B, Q8WYB5), **A** and **B**, respectively. The list of the nearly 70 mammalian species analyzed, and details regarding the precise location of the domain within the sequences can be found in Table S5.

Table S1. GO term enrichment analysis of MR-PrLD and Non-MR-PrLD containing human proteins. The first sheet contains the ID and GO terms

of human proteins containing prion-like domains, sorted as either MR or Non-MR. The second sheet provides the result of a GO term enrichment analysis using as target only the Non-MR-PrLD containing proteins versus the human proteome reference. The third sheet gives the corresponding result but when MR-PrLD containing proteins were used as target. The fourth and fifth sheets contain the GO term enriched specifically in the Non-MR and MR sets, respectively. Finally, the sixth sheet shows the GO terms significantly enriched simultaneously in both sets: MR and Non-MR.

Table S2. Identification and features of human proteins lacking methionine residues.

Table S3. Identification of 78 MR-PrLDs present in 51 different human proteins.

Table S4. List of the Pfam domains accompanying MR-PrLDs.

Table S5. Details regarding MR-PrLDs found in sequences from different mammalian species.
